# How teachers’ social-emotional competence affects bullying in rural schools: the chain mediating effect of students’ social-emotional competence and classmate relationship

**DOI:** 10.3389/fpsyg.2025.1661567

**Published:** 2025-09-04

**Authors:** Jin Ma, Xihao Niu

**Affiliations:** ^1^School of Social Sciences, University of Southampton, Southampton, United Kingdom; ^2^School of Education, Minzu University of China, Beijing, China

**Keywords:** teachers’ social-emotional competence, students’ social-emotional competence, classmate relationship, bullying in rural schools, rural education

## Abstract

**Introduction:**

In recent years, bullying in rural schools has become increasingly prevalent. It is essential to identify the factors that influence bullying in rural schools to better prevent and manage its occurrence.

**Methods:**

This study focuses on Chinese rural students, examining the preventive mechanisms of teachers’ social-emotional competence in mitigating bullying in rural schools. Specifically, the study investigated the mediating role of students’ social-emotional competence and classmate relationship, both individually and as a chain, in this process.

**Results:**

The findings indicated that teachers’ social-emotional competence does not directly reduce bullying in rural schools. Instead, it exerts its influence through the mediating effect of classmate relationship and the chain mediation of “students’ social-emotional competence-classmate relationship.

**Discussion:**

Based on these findings, efforts should focus on improving teachers’ social-emotional competence, enhancing students’ social-emotional competence, and fostering healthier classmate relationship to effectively prevent and control bullying in rural schools.

## 1 Introduction

School bullying refers to the intentional or malicious actions of a student aimed at bullying or insulting other students through physical, verbal, or emotional means (You et al., 2023). Such behavior negatively affects both the immediate and long-term development of students (Brown and Taylor, 2008). Victims of bullying, in particular, often experience decreased academic engagement (Ouellet-Morin et al., 2011), aversion to school (Hutzell and Payne, 2012), and in some cases, early dropout (Cornell et al., 2013). On a broader societal level, students represent the potential human capital of society. Frequent occurrences of school bullying can reduce the accumulation of human capital, thereby hindering the development of economic productivity (Ammermueller, 2012; Ponzo, 2013). In recent years, the prevalence of school bullying has risen in various regions, particularly in rural areas (Wang et al., 2022). Some incidents have even endangered students’ lives. For rural students, especially within the Chinese cultural context, education is a crucial path to improving their life prospects. If students become disengaged from education or drop out due to frequent bullying, it poses a significant threat to their long-term development as well as to the well-being of society. Thus, preventing school bullying in rural schools has become an urgent priority.

Identifying the factors that influence bullying in rural schools is crucial for its prevention (Li H. et al., 2024). Studies have shown that early close relationships, such as attachment with parents, significantly influence an individual’s behavioral development. However, as students transition from home to school, the school environment begins to play an increasingly important role in shaping their behavior (Awiria, 1994). Among the individual students interact with at school, teachers—who have the closest contact with them—play a key role in bullying prevention, particularly through their social-emotional competence. Research indicates that teachers with strong social and emotional skills are better able to empathize with students and teach them how to build effective social relationships, thereby reducing the occurrence of school bullying (Tucker and Maunder, 2015). Thus, teachers’ social-emotional competence is essential in preventing bullying in rural schools.

While many scholars in the field of education have examined the relationship between teachers’ social-emotional competence and school bullying, few have focused on students in rural or underdeveloped areas. Building on previous research, this study investigated the impact of teachers’ social-emotional competence on school bullying and explores the prevention mechanisms specifically in rural areas of China. This study was mainly based on the following research questions:

1. What is the relationship between teachers’ social-emotional competence and bullying in rural schools?
2. Does students’ social-emotional competence play a mediating role in the influence of teachers’ social-emotional competence on bullying in rural schools?
3. Does classmate relationship play a mediating role in the influence of teachers’ social-emotional competence on bullying in rural schools?
4. Do students’ social-emotional competence and classmate relationship play a chain mediating role in the influence of teachers’ social-emotional competence on bullying in rural schools?

## 2 Literature review and hypothesis generation

### 2.1 Research on bullying in rural schools based on attachment theory

Attachment theory provides a theoretical framework for understanding the role of teachers’ social-emotional competence in preventing bullying in rural schools. The theory emphasized the influence of early close relationships, such as parent–child relationships, on individual behavior. However, as students enter the school environment, they form close relationships with teachers, which can be considered secondary attachment relationships. At this stage, the influence of teachers and their social-emotional competence on students’ behavior becomes increasingly significant. Additionally, social-emotional competence, as a valuable internal resource, helps individuals cope with various demands in life and learning, compensating for resource depletion (Zhang et al., 2023). Thus, students’ social-emotional competence plays a crucial role in preventing bullying in rural schools. Furthermore, attachment theory emphasizes the importance of interpersonal relationships in shaping individual behavior and development (Schneider, 1991). These relationships include parent–child, teacher-student, and classmate relationship (Zhang et al., 2024). Since bullying in rural schools primarily occurs among students, classmate relationship is also critical in preventing such behavior. Therefore, grounded in attachment theory, this study focuses on three key factors that influence bullying in rural schools: teachers’ social-emotional competence, students’ social-emotional competence, and classmate relationship.

### 2.2 Teachers’ social-emotional competence and bullying in rural schools

In this study, the definition of teachers’ social-emotional competence aligns with the definition of students’ social-emotional competence outlined in the following section. Since early research on social-emotional competence primarily focused on students, the framework for teachers’ social-emotional competence was developed based on that of students (Zhang, 2021). Additionally, scholars have noted that teachers’ social-emotional competence is, to some extent, consistent with students’ social-emotional competence (Jennings and Greenberg, 2009).

Teachers with strong social-emotional competence can effectively prevent and mitigate school bullying. Related studies have shown that students with poor self-regulation skills are more likely to become “scapegoats” in the classroom and may be subtly provoked by their peers, leading them to engage in aggressive behavior (Jennings and Greenberg, 2009). Teachers with this competence is better able to perceive and address these situations effectively (Jennings and Greenberg, 2009). In other words, such teachers can help prevent school bullying to a certain extent. Additionally, an empirical study investigating the role of teachers’ social-emotional competence in students’ beliefs about school bullying supported this view (Garner, 2017). Therefore, teachers with strong social-emotional abilities can play a significant role in preventing bullying in rural schools. Based on this, research hypothesis 1 is proposed.

*H1*: Teachers’ social-emotional competence negatively predicts bullying in rural schools.

### 2.3 The mediating role of students’ social-emotional competence

Teachers’ social-emotional competence can help prevent bullying in rural schools by enhancing students’ social-emotional competence. Students’ social-emotional competence refers to their ability to interact with others, monitor and regulate cognitive processes, and manage emotions and behaviors (Ahmed et al., 2020). Scholars typically divided it into five components: responsible decision-making, relationship management, social awareness, self-awareness, and self-management (Mantz et al., 2018). Chinese researchers, incorporating international findings, approached social-emotional competence from a process perspective, dividing it into “three relationships and six dimensions.” These include building relationships with oneself (self-cognition and self-management), building relationships with others (social cognition and interpersonal relationships), and building relationships with the collective (collective cognition and collective management) (Du and Mao, 2018).

On the one hand, teachers’ social-emotional competence contributes to the improvement of students’ social-emotional competence, as supported by relevant research. Theoretical studies suggested that teachers with higher social-emotional competence are more effective in delivering courses that cultivate these skills and promote the development of students’ social-emotional competence through modeling. Such teachers also enhance students’ social-emotional learning through effective classroom management and by fostering a positive classroom atmosphere (Jennings and Greenberg, 2009), thereby improving students’ social-emotional competence. Additionally, empirical studies have shown that teachers’ social-emotional competence positively impact students’ social-emotional competence (Li et al., 2021), and that teachers’ social-emotional beliefs play a crucial role in this process (Zhang and Mao, 2020). On the other hand, students’ social-emotional competence can effectively prevent bullying in rural schools. An empirical study of primary school students in Beijing found that students’ social-emotional competence negatively predicts campus bullying behavior, with the “other management” sub-dimension of social-emotional competence being more strongly correlated with bullying behavior than other dimensions (Du et al., 2018). Furthermore, a quasi-experimental study conducted by Korean scholars to prevent adolescent bullying supports this view. The results showed that students who received social-emotional competence education not only significantly improved their social-emotional competence but also experienced a substantial reduction in the frequency of bullying behaviors (Song and Kim, 2022). This evidence demonstrates that students’ social-emotional competence can effectively prevent school bullying. Therefore, teachers’ social-emotional competence can prevent bullying in rural schools by enhancing students’ social-emotional abilities. Based on this, research hypothesis 2 is proposed.

*H2*: Students’ social-emotional competence plays a mediating role between teachers’ social-emotional competence and bullying in rural schools.

*H2a*: Teachers’ social-emotional competence positively predicts students’ social-emotional competence.

*H2b*: Students social-emotional competence negatively predicts bullying in rural schools.

### 2.4 The mediating role of classmate relationship

Teachers’ social-emotional competence can help prevent bullying in rural schools by fostering positive classmate relationship. Drawing on Longobardi et al. (2016) definition of teacher-student relationship, we define classmate relationship as meaningful emotional and relational connections formed between students through long-term interactions. Unlike relationships formed by adults, such as parent–child or teacher-student relationships, classmate relationship is unique because they are voluntary and, in principle, horizontal (Endedijk et al., 2022).

On the one hand, teachers’ social-emotional competence helps improve classmate relationship, as supported by relevant research. Studies have shown that teachers with this competence demonstrate more advanced social-emotional skills. By learning from such teachers, students are encouraged to show greater empathy and build and maintain positive relationships with others (Loukatari et al., 2019). Additionally, an empirical study explains this further by examining student gender, revealing that teachers positively impact classmate relationship, though this effect is stronger for girls. This is because teachers tend to invest more emotional labor when interacting with girls, helping them communicate with peers through comfort and redirection (Garner and Legette, 2023). On the other hand, positive classmate relationship can also effectively prevent bullying in rural schools. An empirical study based on social cognitive theory explored adolescents’ willingness to prevent school bullying and found that both teacher-student and classmate relationship are directly related to students’ intervention in bullying (Wachs et al., 2020). In other words, strong classmate relationship encourages students to take the initiative in stopping bullying. Other research focusing on social skills shows that these skills negatively predict bullying behavior because they foster positive interactions with peers, thereby reducing bullying (Trigueros et al., 2020). A longitudinal study further supported this view, showing that classmate relationship interacts with bullying behavior. Supportive relationships influence the social dynamics between bullies and victims, reducing the occurrence of bullying (Healy et al., 2024). Therefore, teachers’ social-emotional competence can help prevent bullying in rural schools by fostering stronger peer relationships. Based on this, research hypothesis 3 is proposed.

*H3*: Classmate relationship plays a mediating role between teachers’ social-emotional competence and bullying in rural schools.

*H3a*: Teachers’ social-emotional competence positively predicts classmate relationship.

*H3b*: Classmate relationship negatively predicts bullying in rural schools.

### 2.5 Chain mediating effect of students’ social-emotional competence and classmate relationship

The impact of students’ social-emotional competence on classmate relationship has also gained attention in the academic community. Studies have shown that classmate relationship is among the most important interpersonal connections for students, and social-emotional competence is a key factor influencing the dynamics of these relationships (Li X. et al., 2024). Since social-emotional competence is crucial throughout life for achieving social outcomes, they contribute to the attainment of personal goals, enhance autonomy, and foster harmonious interpersonal relationships (OECD, 2015). Therefore, students’ social-emotional competence naturally has a positive effect on the development of strong classmate relationship. Additionally, an empirical study on primary and secondary school students supports this view, showing that students with higher social-emotional competence possess stronger social skills, are better able to perceive the emotions of others, and can effectively resolve conflicts in peer interactions, thereby establishing positive classmate relationship (You et al., 2023).

In summary, considering the separate mediating roles of students’ social-emotional competence and classmate relationship, the “students’ social-emotional competence–peer relationship” plays a chain mediating role in preventing and addressing rural school bullying through teachers’ social-emotional competence. This indicates that teachers’ social-emotional competence can enhance students’ social-emotional competence, which in turn improves classmate relationship and ultimately helps prevent bullying in rural school. Based on this, the study proposes research hypothesis 4.

*H4*: Students’ social-emotional competence and classmate relationship play a chain mediating role in the process in which teachers’ social-emotional competence affect bullying in rural schools.

*H4a*: Students’ social-emotional competence positively predicts classmate relationship.

**Figure 1:**
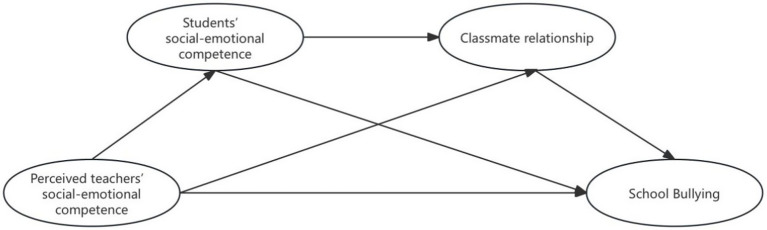
The hypothesized research model.

Based on this, the study constructs a chain mediation model, where teachers’ social-emotional competence serves as the predictor variable, bullying in rural schools as the outcome variable, and students’ social-emotional competence and classmate relationship as mediating variables (see Figure 1).

## 3 Methods

### 3.1 Participants

This study surveyed students from a rural school in Shandong Province, using offline paper questionnaires to collect data from 535 respondents. After excluding 8 invalid responses, 527 valid questionnaires remained, yielding an effective response rate of 98.5%. The sample included 276 boys (52.3%) and 251 girls (47.6%), with 253 students in grade 8 (48%) and 274 students in grade 9 (51.9%). Additionally, 246 students (46.6%) had class leadership experience, 55 students (10.4%) were left-behind children, and 82 students (15.5%) were only children.

### 3.2 Instruments

This study utilized the Students’ Perception of Teachers’ social-emotional Competence Scale, the Students’ social-emotional Competence Scale, the Peer Support Scale, and the Peer Aggression Scale to conduct the investigation and research.

#### 3.2.1 Teachers’ social-emotional competence

Given that the participants of this study were rural students, the Student Perception of Teachers’ social-emotional Competence Scale developed by Whitehead (2013) was used to assess teachers’ social-emotional competence. The original scale has six items, but during the confirmatory factor analysis (CFA), we found that the factor loading of two of the items did not reach the commonly used threshold (<0.5), indicating that the relationship between these items and the latent variables was weak and could not well reflect the construct to be measured. In order to ensure the convergent validity and discriminant validity of the measurement model, we eliminated these two items according to statistical standards and retained only four items with qualified factor loading, such as, “My teacher seems very happy this year. My teacher really cares about whether students understand what he or she is teaching. My teacher really enjoys teaching our class. My teacher pays close attention to students’ emotional changes in class, especially when someone feels sad or anxious.” The rural students participating in the survey rated their teachers’ social-emotional competence based on their everyday perceptions, using a scale ranging from 1 (completely disagree) to 5 (completely agree). Higher scores indicated a greater level of the teachers’ social-emotional competence.

#### 3.2.2 Students’ social-emotional competence

The students’ social-emotional competence was primarily measured using the Student social-emotional Competence Scale developed by Mantz et al. (2018). The scale comprises four dimensions: responsible decision-making, relationship skills, self-management, and social awareness. Each dimension includes three items, such as, “When I get into trouble, I never blame others.” The rural students participating in the survey assessed themselves based on their personal experiences, with responses ranging from 1 (completely disagree) to 5 (completely agree). Higher scores indicated stronger social-emotional competence among the rural students. Because there are forward-scored items in this scale, we first use the forward-scoring method to process the data before analyzing it.

#### 3.2.3 Classmate relationship

The between classmate relationship was primarily assessed using the Peer Support Scale developed by Torsheim et al. (2012). The scale consists of four items, such as, “When a classmate feels depressed, other classmates do their best to help and comfort them.” The rural students participating in the survey provided quantitative assessments based on their typical interactions with classmates, using a scale from 1 (completely disagree) to 5 (completely agree). Higher scores indicated stronger peer relationships.

#### 3.2.4 Bullying in rural schools

Bullying in Rural school was primarily assessed using the Peer Aggression Scale developed by Wang et al. (2011). The original scale has eight items, but during the CFA, we found that the factor loading of four of the items did not reach the commonly used threshold (<0.5), indicating that the relationship between these items and the latent variables was weak and could not well reflect the construct to be measured. In order to ensure the convergent validity and discriminant validity of the measurement model, we eliminated these four items according to statistical standards and retained only four items with qualified factor loading, such as, “Other students bullied me, beat me, or locked me in the room. Other students deliberately excluded me or ignored me completely. Other students spread rumors about me, trying to make others dislike me. Other students spread rumors about me through text messages.” The rural students participating in the survey rated their experiences based on their actual situations, using a scale from 1 (never) to 5 (always). Lower scores indicated less frequent occurrences of bullying in rural schools.

### 3.3 Data analysis

This study primarily employed two software packages, SPSS 26 and Mplus8.3. First, SPSS 26 was used to test for common method bias, perform descriptive statistics, and conduct correlation analysis of the data. Next, Mplus8.3 was utilized to assess the measurement model and to construct and analyze the chain mediation model.

## 4 Results

### 4.1 Examination of the measurement model

Firstly, reliability and validity analysis was conducted on teachers’ social-emotional competence, students’ social-emotional competence, classmate’ relationship, and bullying in rural schools. As shown in Table 1, the standardized factor loading of all questions ranged from 0.576 to 0.934. The combined reliability of each variable exceeded the critical value of 0.7. The average variance extraction exceeded the critical value of 0.5, indicating that there was a good correspondence between the factors and the measurement items, and the convergent validity was good. The Cronbach’s α coefficient of each measurement variable was higher than 0.7, indicating that the measurement model had good internal consistency. Secondly, the fitting validity test was conducted on teachers’ social-emotional competence, students’ social-emotional competence, classmate’ relationship and bullying in rural schools. The results showed that teachers’ social-emotional competence (χ^2^ = 9.210, df = 2, χ^2^/df = 4.605, CFI = 0.990, TLI = 0.969, RMSEA = 0.083, SRMR = 0.017), students’ social-emotional competence (χ^2^ = 174.410, df = 50, χ^2^/df = 3.488, CFI = 0.962, TLI = 0.950, RMSEA = 0.069, SRMR = 0.032), classmate’ relationship (χ^2^ = 4.329, df = 2, χ^2^/df = 2.165, CFI = 0.998, TLI = 0.994, RMSEA = 0.047, SRMR = 0.016), and bullying in rural schools were significantly correlated with teachers’ social-emotional competence (χ^2^ = 9.210, df = 2, χ^2^/df = 4.605, CFI = 0.990, TLI = 0.969, RMSEA = 0.083, SRMR = 0.017). SRMR = 0.008 and bullying in rural schools (χ^2^ = 8.186, df = 2, χ^2^/df = 4.093, CFI = 0.991, TLI = 0.972, RMSEA = 0.077, SRMR = 0.017) all have good fitting results. Finally, the discriminant validity of the measurement model was tested, as shown in Table 2. The values on the diagonal are greater than other values in the same line or column, indicating that there is good discriminant validity between the measurement models. In summary, our measurement model supports further analysis.

**Table 1:** Reliability and validity test results.

Measurement model	Factor loading	CR	AVE	α
Teachers’ social-emotional competence	0.600–0.799	0.812	0.522	0.805
Students’ social-emotional competence	0.787–0.934	0.933	0.778	0.870
Classmate relationship	0.773–0.882	0.887	0.664	0.885
Bullying in rural schools	0.576–0.840	0.799	0.504	0.774

CR, Combined reliability; AVE, Average Variance Extraction.

**Table 2:** Discriminant validity.

Measurement model	1	2	3	4
1. Teachers’ social-emotional competence	0.722			
2. Students’ social-emotional competence	0.677***	0.882		
3. Classmate relationship	0.674***	0.733***	0.8149	
4. Bullying in rural schools	−0.224***	−0.255***	−0.344***	0.710

***p < 0.001.

### 4.2 Descriptive statistics and correlation analysis

Descriptive statistics and correlation analysis were conducted on teachers’ social-emotional competence, students’ social-emotional competence, classmate’ relationship, and bullying in rural schools. As shown in Table 3, teachers’ social-emotional competence (*M* = 4.060 > 3, *p* < 0.001), students’ social-emotional competence (*M* = 4.044 > 3, *p* < 0.001), and classmate’ relationship (*M* = 4.130 > 3, *p* < 0.001) were all at an upper-middle level, and bullying in rural schools was at a lower-middle level (*M* = 1.131 < 3, *p* < 0.001). At the same time, teachers’ social-emotional competence was significantly positively correlated with students’ social-emotional competence and classmate’ relationship (*p* < 0.001), and was significantly negatively correlated with bullying in rural schools (*p* < 0.001); students’ social-emotional competence was significantly positively correlated with classmate’ relationship (*p* < 0.001), and was significantly negatively correlated with bullying in rural schools (*p* < 0.001); classmate’ relationship were significantly negatively correlated with bullying in rural schools (*p* < 0.001).

**Table 3:** Correlation matrix of main variables.

Variables	*M* ± SD	1	2	3	4
1. Teachers’ social-emotional competence	4.060 ± 0.695	1			
2. Students’ social-emotional competence	4.044 ± 0.598	0.677***	1		
3. Classmate relationship	4.130 ± 0.704	0.674***	0.733***	1	
4. Bullying in rural schools	1.131 ± 0.339	−0.224***	−0.255***	−0.344***	1

M, Mean; SD, Standard deviation. ***p < 0.001.

### 4.3 Comparison of complex and nested models

To test the effect of the chain mediation model, this study controlled variables such as rural students’ gender, grade, class leadership experience, whether they were left-behind children, and whether they were only children, and compared several nested models.

We first constructed a complex model, specifically a chain mediation model, with teachers’ social-emotional competence as the predictor variable, bullying in rural schools as the outcome variable, and students’ social-emotional competence and classmate’ relationship as mediating variables. Afterwards, we constructed four nested models by constraining specific paths to zero. For example, nested model 1 was based on the complex model, but excluded the path from students’ social-emotional competence to classmates’ relationships; nested model 2 was based on the complex model, but excluded the path from classmate’ relationship to bullying in rural schools; nested model 3 was based on the complex model, but excluded the path from students’ social-emotional competence to bullying in rural schools; and nested model 4 was based on the baseline model, but excluded the path from classmate’ relationship to bullying in rural schools and the path from students’ social-emotional competence to bullying in rural schools.

As shown in Table 4, nested model 3 has the best fit, indicating that deleting the path of “students’ social-emotional competence → bullying in rural schools” can improve the model fit. Therefore, in subsequent analyses, we will only focus on nested model 3, which shows that students’ social-emotional competence has no independent mediating effect. Based on this description, hypothesis 2 was not confirmed.

**Table 4:** Goodness of fit of complex and nested models.

Model type	χ^2^	df	CFI	TLI	RMSEA	SRMR
Complex model	805.947	342	0.934	0.923	0.051	0.038
Nested model 1	848.481	343	0.928	0.916	0.053	0.042
Nested model 2	825.256	343	0.931	0.920	0.052	0.040
Nested model 3	806.661	343	0.934	0.923	0.051	0.038
Nested model 4	827.467	344	0.931	0.920	0.052	0.041

### 4.4 The chain mediation effect test

**Figure 2:**
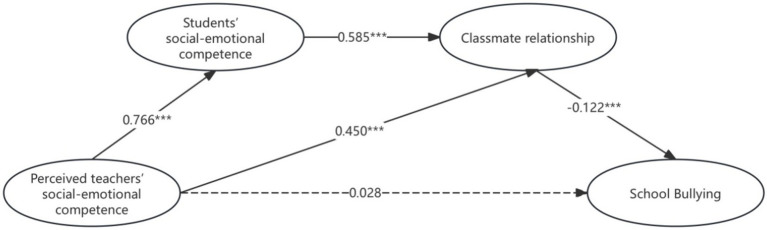
The chain mediation model.

The chain mediation model was tested for mediation effect. As shown in Figure 2, teachers’ social-emotional competence significantly and positively predicted students’ social-emotional competence (β = 0.766, *p* < 0.001) and classmate’ relationship (β = 0.450, *p* < 0.001), students’ social-emotional competence significantly and positively predicted classmate’ relationship (β = 0.585, *p* < 0.001), and classmate’ relationship significantly and negatively predicted bullying in rural schools (β = −0.122, *p* < 0.001).

Furthermore, the Bootstrap method was used to further test the mediation effect. Repeated sampling was used 5,000 times to test whether the single mediation effect of classmate relationship and the chain mediation effect of students’ social-emotional competence and classmate relationship were significant. The main observation was whether the 95% confidence interval (95%CI) contained 0. If it did not contain 0, it would indicate that the single mediation effect and the chain mediation effect were significant. As shown in Table 5 and Figure 2, teachers’ social-emotional competence indirectly prevented bullying in rural schools through two mediation paths, namely, path 1 “teachers’ social-emotional competence → classmate relationship → bullying in rural schools,” the path effect value was −0.055, 95%CI was (−0.134, −0.023), path 2 “teachers’ social-emotional competence → students’ social-emotional competence → classmate relationship → bullying in rural schools,” the path effect value was −0.055, 95%CI was (−0.118, −0.023).

**Table 5:** Mediation effect size estimates.

Path	Point estimate	S. E.	*P*	95%CI		Hypothesis
				Lower	Upper	
Total effect	−0.082	0.027	0.003	−0.149	−0.039	–
Direct effect	0.028	0.041	0.503	−0.039	0.130	Hypothesis 1 was not supported
Total indirect effect	−0.110	0.042	0.009	−0.228	−0.049	–
Path1	−0.055	0.025	0.030	−0.134	−0.023	Hypothesis 3 is supported
Path 2	−0.055	0.022	0.013	−0.118	−0.023	Hypothesis 4 is supported

## 5 Discussion

### 5.1 The current situation

This study found that the mean score for bullying in rural schools was less than the median of 3, indicating that bullying was at a moderately low level in the rural schools we surveyed. This may be because, compared to urban schools, rural schools are typically smaller, with a limited student population, and students are familiar with each other and interact frequently (Wang et al., 2024). This “acquaintance society” reduces anonymity, making bullying easier to detect and stop. Secondly, rural schools may also have lower student-teacher ratios, allowing teachers to pay more attention to their students, which helps to curb serious bullying (Liu et al., 2024; Wang et al., 2024). Finally, considering traditional Chinese values that emphasize collectivism, mutual assistance, and obedience, rural students are often influenced by these values (Liu et al., 2025). Therefore, based on this, bullying in rural schools may be somewhat suppressed.

Furthermore, this study found that the mean scores for rural teachers’ social-emotional competence, students’ social-emotional competence, and classmate relationship were all above the median of 3, indicating that the teachers’ social-emotional competence, students’ social-emotional competence, and classmate relationship of the rural schools we surveyed were all above average, consistent with previous research (Hoferichter et al., 2022; Wang et al., 2019; Zhang et al., 2023). This may be because teachers in this rural school place greater emphasis on emotional communication and the maintenance of teacher-student relationships in daily teaching and classroom management. Due to the smaller school size and frequent teacher-student contact (Liu et al., 2024; Wang et al., 2024), teachers are more likely to address students’ psychological well-being and emotional needs in daily teaching and daily life (Michaud and Montreuil, 2025), thereby subtly enhancing their own social-emotional competence. Furthermore, rural students grow up in relatively stable and close-knit interpersonal networks, resulting in a high degree of familiarity and more direct and natural interactions, which helps them develop strong social-emotional competence through interaction. At the same time, rural schools generally have harmonious peer relationships and a strong sense of community, making it easier for students to establish positive interpersonal connections in an environment of mutual support and cooperation, resulting in a high level of overall peer relationships (Fargas Malet and Bagley, 2024; Gristy, 2012). This may also be related to the increasing emphasis placed by the government, society, and schools in recent years on the cultivation and development of students’ and teachers’ social-emotional competence. The government has introduced a series of policies and provided substantial policy and material support. For example, the “Interim Implementation Measures for the Accreditation of Normal Education Programs in Regular Institutions of Higher Educatio” issued by the Ministry of Education of China in October 2017 outlined training objectives for pre-service teachers by graduation. These objectives encompass four dimensions: practicing professional ethics, learning to teach, learning to educate, and learning to develop. These objectives address social-emotional competence, including teaching ability, comprehensive education, learning to reflect, and communicating and collaborating.

However, it is worth noting that the results of this study are based on a survey of a single rural school. While of some reference value, their representativeness is limited and cannot fully reflect the true situation of all rural schools. Therefore, the generalizability of the findings should be viewed with caution. Future research could include surveys in more regions and across different types of rural schools to enhance the generalizability and external validity of the results.

### 5.2 The impact of teachers’ social-emotional competence on bullying in rural schools

The results of this study showed that, in a bivariate correlation analysis, teacher” social-emotional competence was significantly negatively correlated with bullying in rural schools. However, after incorporating students’ social-emotional competence and classmate relationship into the mediation analysis, the direct effect of teachers’ social-emotional competence on school bullying was no longer significant, while the indirect effect was significant, indicating that Hypothesis 1 was not confirmed (Garner, 2017). This result is consistent with a complete mediation model, indicating that teachers’ social-emotional competence reduces bullying behavior primarily through improving students’ social-emotional competence and fostering positive classmate relationship.

This may be because teachers’ social-emotional competence is an external environmental trait, and its main mechanism of action is to indirectly influence students’ bullying behavior by improving their social-emotional competence and creating a positive classmate relationship atmosphere (Poulou and Denham, 2023; You et al., 2023). Secondly, according to ecological systems theory, the proximal determinants of bullying behavior are primarily at the individual level (such as empathy) and the peer level (such as classmate relationship) (Espelage, 2014; Lambe et al., 2019), while teachers’ social-emotional competence represents a higher-level contextual resource. Therefore, the effect of teachers’ social-emotional competence on school bullying appears to be “complete mediation,” in which the influence is indirect through students and peer relationships, rather than directly affecting students’ bullying behavior.

### 5.3 Mediating role of students’ social-emotional competence

This study found that students’ social-emotional competence does not play a significant mediating role between teachers’ social-emotional competence and bullying in rural schools, and hypothesis 2 was not confirmed.

On the one hand, teachers’ social-emotional competence positively influences students’ social-emotional competence, further validated previous research (Alzahrani et al., 2019). In daily teaching, teachers with this competence naturally model these skills and subtly impart related knowledge and abilities to rural students, resulting in improved students’ social-emotional competence. This is particularly important in rural areas, where, due to challenging economic conditions, many parents work away from home, often neglecting their children’s emotional education (Shi et al., 2021). Consequently, the role of parents as emotional role models is diminished. In this context, teachers—who are among the closest adults to rural students—become especially influential. Rural students often view their teachers as “authoritative” figures to imitate, and as a result, teachers’ social-emotional competence plays a crucial role in shaping students’ social-emotional competence. On the other hand, the path of the impact of students’ social-emotional competence on bullying in rural schools is not valid, which differed from previous research findings (Yang et al., 2020). This may be due to rural areas are “acquaintance-based” societies (Li et al., 2023), making bullying not just a conflict between students but also potentially involving family and neighborhood relationships. Rural students’ social-emotional competence is less able to withstand this “network pressure.” Secondly, bullying behavior is often more directly influenced by peer relationship and group norms (Duffy and Nesdale, 2009), while rural students’ social-emotional competence is an “internal resources” (Collie et al., 2024). Therefore, peer pressure and group relationships may overwhelm the impact of rural students’ social-emotional competence. Therefore, under these conditions, teachers’ social-emotional competence does not effectively prevent bullying in rural schools through the enhancement of students’ social-emotional competence.

### 5.4 Mediating role of classmate relationship

This study found that classmate relationship plays a significant mediating role between teachers’ social-emotional competence and bullying in rural schools. This result confirmed Hypothesis 3.

On the one hand, teachers’ social-emotional competence has a positive influence on classmate relationship, further supported previous research (Garner, 2010). Such teachers tend to exhibit higher social skills (Gimbert et al., 2023) and are better equipped to manage relationships with both colleagues and students. The students in this study are in adolescence, a stage where imitation is a key learning method. As a result, they often model their interactions with classmates on the way teachers interact with others, thereby fostering positive classmate relationship. Additionally, when teachers possess strong social-emotional competence, they shape students’ perspectives on their peers, encouraging them to notice and appreciate the strengths and positive qualities in their classmates (Morgan et al., 2022). This promotes a willingness to engage in meaningful communication, which helps to strengthen peer relationships. On the other hand, good classmate relationship can effectively prevent bullying in rural schools, further supported previous studies (Van Ryzin and Roseth, 2018). Bullies often target students who lack social connections in their peer group to avoid losing the approval of important peers (Veenstra et al., 2010). In rural areas, students without strong classmate relationship are more vulnerable to bullying. Friends provide an essential source of affection and belonging, helping students maintain their social standing (Sentse et al., 2013). Those with many friends receive higher levels of social support and peer recognition (Gazelle et al., 2023), indicating well-developed classmate relationship. In contrast, students with few or no friends are more likely to be excluded and become targets of bullying (Pedersen et al., 2007). Therefore, teachers’ social-emotional competence can help prevent bullying in rural school by fostering stronger classmate relationship.

### 5.5 The chain mediating effect of students’ social-emotional competence and classmate relationship

The results of the chain mediation test indicate that “students’ social-emotional ability–classmate relationship” played a mediating role in preventing bullying in rural school through the influence of teachers’ social-emotional competence, confirming Hypothesis 4. Specifically, students’ social-emotional competence positively affected classmate relationship, further validated previous research (Taylor et al., 2017). Consistent with prior studies (Zhang et al., 2023), the foundation of strong classmate relationship is built on trust, respect, and understanding. Students with higher levels of social-emotional competence exhibit greater empathy, which enables them to view situations from others’ perspectives and show more tolerance and understanding in their interactions with classmates (Imuta et al., 2022). Furthermore, as supported by earlier research rural students with higher social-emotional competence are better able to regulate their emotions, control anger, and resolve conflicts with friends, thus maintaining healthy and reciprocal classmate relationship (Wang et al., 2019). Consequently, teachers’ social-emotional competence enhances students’ social-emotional competence, which in turn improves rural students’ classmate relationship, ultimately contributing to the prevention of school bullying.

Attachment theory emphasized that school-based interpersonal relationships consist of both teacher–student and classmate relationship, which jointly shape students’ social and emotional development as well as their behavioral outcomes (Cassidy et al., 1996; Verschueren and Koomen, 2012). In line with this framework, our findings suggest that teachers’ social-emotional competence not only serves as an individual professional capacity, but also functions as an important determinant of the quality of teacher–student interactions. When teachers demonstrate high levels of social-emotional competence, students are more likely to experience emotional security, develop stronger social-emotional competence, and build positive classmate relationship. These processes, in turn, contribute to a reduction in school bullying. Thus, although our model explicitly examined the mediating role of students’ social-emotional competence and classmate relationship, the results can also be interpreted as reflecting the underlying influence of teacher–student relationship, consistent with the principles of attachment theory.

## 6 Implications

This study holds both theoretical and practical significance. Theoretically, it focuses on rural areas, examining the mechanisms for preventing school bullying from a rural perspective. This not only offers valuable insights for educational management in underdeveloped regions but also broadens the scope of research in this field. Additionally, while previous studies have explored school bullying prevention mechanisms, there has been a lack of attention to the role of teachers’ social-emotional competence. By addressing this gap, the present study contributes to the literature by emphasizing the importance of teachers’ social-emotional competence and introduces a new perspective on bullying prevention, particularly in rural and underdeveloped areas.

Practically, educators and administrators should focus on enhancing teachers’ social-emotional competence, improving students’ social-emotional competence, and fostering positive classmate relationships to prevent bullying in rural schools. First, teachers should prioritize fostering a positive classroom climate and promoting healthy classmate relationships by cultivating social-emotional competence during daily teaching activities. Training teachers to recognize and regulate their own emotions, understand students’ emotions, and actively deliver social-emotional education within the classroom can have a beneficial impact on bullying prevention. Second, schools and educational administrators should emphasize the development of students’ social-emotional competence. Through curriculum design, extracurricular activities, and home–school collaboration, they can jointly enhance students’ emotional regulation and interpersonal skills, thereby supporting the establishment of harmonious classmate relationship and fundamentally reducing bullying. Finally, given the educational context of underdeveloped rural areas, it is recommended to formulate more targeted teacher training initiatives and policies supporting students’ social-emotional development. Integrating the cultivation of teachers’ social-emotional competence with the improvement of students’ classmate relationship can help build a systematic anti-bullying mechanism. This approach will not only improve the quality of rural school education, but also provide a solid talent foundation and social-emotional support for advancing educational equity and promoting rural revitalization.

## 7 Limitations and suggestions

This study has certain limitations due to time and scope constraints. First, because of the time limitations, the cross-sectional design employed in this research does not provide a detailed understanding of how teachers’ social-emotional competence, students’ social-emotional competence, and classmate relationship influence rural school bullying over time. Future research could adopt a longitudinal approach to explore these relationships more thoroughly, ensuring greater rigor in the data and results. Second, our study sampled only 527 students from a single rural school in Shandong Province. While representative, this sample does not adequately reflect the overall situation in rural schools across China, and the conclusions should be generalized with caution. Future research should be based on a larger, more cross-regional sample, and incorporate rural schools from diverse economic and cultural backgrounds to enhance the external validity of the findings. Finally, our measurement of teachers’ social-emotional competence did not directly measure class teachers’ self-assessment, but rather indirectly measured students’ perceptions of their teachers’ social-emotional competence. Furthermore, students’ social-emotional competence were based on students’ own assessments. Students’ assessments may be inflated due to implicit pressure on teachers and their own desire to save face. Therefore, further research could combine class teachers’ and students’ self-assessments with independent observations, or expand the research population, for example by including school leaders or parents, to obtain more comprehensive measurement results.

## 8 Conclusion

This study is based on rural students, conducting a comprehensive analysis of the preventive mechanisms of teachers’ social-emotional competence in addressing bullying in rural schools. The key findings revealed that teachers’ social-emotional competence does not directly prevent bullying in rural schools. Instead, it exerts its influence through the mediation of classmate relationship and through a chain mediation involving students’ social-emotional competence and classmate relationship. Overall, this study offers valuable insights into preventing bullying in rural schools, particularly in underdeveloped areas. These findings also serve as a useful reference for educators and policymakers in managing students and formulating policy recommendations.

## Data Availability

The original contributions presented in the study are included in the article/supplementary material, further inquiries can be directed to the corresponding author.
